# Assessment of the effects of rapid diagnostic biofire blood culture identification panel in hospitalized patients

**DOI:** 10.1017/ash.2022.140

**Published:** 2022-05-16

**Authors:** Amy Cohen, Thomas Erwes, Dora Wiskirchen, Jessica Abrantes-Figueiredo

## Abstract

**Background:** Bloodstream infections (BSIs) have life-threatening consequences; they contribute to increased global morbidity and mortality, particularly in critically ill patients. Consequently, early implementation of effective antimicrobial therapy is crucial. Microbiology stewardship efforts, such as rapid diagnostic testing, streamline healthcare resources while also optimizing clinical outcomes. These outcomes include decreased mortality, fewer days of hospitalization, and more efficient time to appropriate anti-infective regimens. Biofire Blood Culture Identification (BCID) is a 2-stage multiplexed PCR system yielding multiple pathogen etiologies, as well as antimicrobial resistance genes. Results are published ~60 minutes after a blood-culture Gram stain turns positive. The purpose of this study was to assess the clinical impact of rapid diagnostic PCR testing, which was introduced at Saint Francis Hospital in March 2020. **Methods:** We conducted a single-center, retrospective observational chart review before and after implementation of Biofire BCID, surveying all positive cultures from December 2019 through June 2020. Medical records were more thoroughly reviewed for patients who met study inclusion criteria. The primary outcome of interest, time to appropriate antimicrobial therapy, included both days to targeted therapy in the setting of a probable pathogen, and days to antibiotic discontinuation in the case of a likely contaminant (nonpathogenic normal skin flora introduced into culture at the time of collection or processing). Secondary outcomes included in-hospital mortality (death during hospitalization), and inpatient length of stay (LOS). Wilcoxon rank-sum tests were used for primary outcomes and Fisher exact tests were used for secondary outcomes. **Results:** Among 643 patients with positive blood cultures, 410 (63.8%) met the criteria. In the study, 220 patients before the intervention and 190 patients after the intervention were reviewed. The difference in mean days to targeted therapy with a probable pathogen and days to antibiotic discontinuation with a likely contaminant were both observed at a significance level (3.62 vs 1.79, P Inpatient mortality rates were higher prior to launching Biofire BCID, but they were not statistically significant (15.5% vs 14.2 %; *P* = .782). The average LOS before and after implementation was 12.6 days (range, 2–92 days), and 10 days (range, 2–68 days), respectively. This parameter was also not statistically significant (*P* = .597). **Conclusions:** We detected a trend toward a significant reduction in time to appropriate antimicrobial therapy following the launch of Biofire BCID. Incorporation of molecular rapid diagnostics for BSI evaluation should be the standard of care in hospital settings.

**Funding:** None

**Disclosures:** None

